# Measurements and Digital Technology Solutions to Monitor Physical Activity in Patients With Pediatric Cancer: Scoping Review

**DOI:** 10.2196/73889

**Published:** 2026-01-29

**Authors:** Greta Franceska Jermolenko, Guna Semjonova, Aija Klavina, Evita Dubinina, Keita Augstkalne, Klavs Balamovskis-Kalnins, Alina Cesuna, Emil Syundyukov, Dace Bertule, Madara Blumberga, Ilze Kundzina

**Affiliations:** 1 Laboratory of Sports and Nutrition Research Riga Stradins University Riga Latvia; 2 Latvian Academy of Sport Education Riga Stradiņš University Riga Latvia; 3 Department of Rehabilitation Rīga Stradiņš University Riga Latvia; 4 Department of Health Promotion and Rehabilitation Lithuanian Sports University Riga Latvia; 5 Klaipėdos Valstybinė Kolegija/ Higher Education Institution Klaipeda Lithuania; 6 Faculty of Medicine and Life Sciences University of Latvia Riga Latvia; 7 Children's Clinical University Hospital Riga Latvia; 8 Medical Education Technology Center Rīga Stradiņš University Riga Latvia; 9 Joint study program Service Design Strategies and Innovation Art Academy of Latvia Riga Latvia

**Keywords:** pediatric oncology, cancer, physical activity monitoring, augmented reality, digital health solutions, gamification

## Abstract

**Background:**

Patients with pediatric cancer often experience reduced physical activity (PA) due to treatment-related fatigue, functional limitations, and lack of structured exercise programs. Digital health solutions, including wearable sensors and augmented reality (AR)-based interventions, may offer new possibilities for monitoring and improving PA in this population.

**Objective:**

This scoping review aims to address existing research gaps by identifying the instruments—both conventional and digital—used to monitor PA in patients with pediatric cancer during treatment. In addition, this study examines PA monitoring methods, identifies the variables collected, and explores the applicability of digital health solutions in facilitating PA engagement among patients with pediatric cancer.

**Methods:**

In accordance with the Joanna Briggs Institute methodology, a systematic search was conducted across 8 scientific databases—ProQuest, Web of Science, EBSCO Complete, Google Scholar, ScienceDirect, Scopus, MEDLINE (PubMed), and Cochrane—on April 18 and 19, 2024. Studies were screened using the Rayyan AI-assisted review tool based on predefined inclusion criteria targeting children aged 7-19 years who were undergoing cancer treatment or were within 2 years posttreatment. Eligible studies included clinical trials and observational studies that examined objective (eg, wearable sensors) and subjective (eg, questionnaires and self-reports) approaches to PA monitoring. Keywords and controlled vocabulary (eg, MeSH [Medical Subject Headings] terms) were identified through a review of relevant literature. Data were extracted systematically to capture study characteristics, intervention types, and outcome measures. Extracted data were charted and synthesized narratively to identify patterns, technological applications, and research gaps in PA monitoring among patients with pediatric cancer.

**Results:**

Twelve studies met the inclusion criteria and employed a range of PA monitoring tools. Digital health solutions, including Actical and Garmin VivoFit 3 devices, were used in 5 studies to assess step counts, gait cycles, and movement intensity. Self-reported measures were identified in 11 studies, most commonly the Activities Scale for Kids and the Pediatric Quality of Life Inventory-Multidimensional Fatigue Scale, which provided insights into mobility and fatigue. Despite their feasibility, subjective assessments were limited by recall bias and motivational factors. Although digital health solutions—such as wearable sensors, gamification, and mobile applications—showed potential to improve PA adherence, their application remains underutilized, and evidence regarding their integration in pediatric oncology is limited.

**Conclusions:**

Existing objective and subjective methods for monitoring PA provide valuable insights; however, gaps remain in the use of interactive digital health solutions, such as AR-based interventions, for PA monitoring and engagement. Future research should focus on integrating digital tools that not only track PA but also actively engage patients, enhance motivation, and support rehabilitation across both clinical and home settings.

## Introduction

Every year, approximately 400,000 children and adolescents aged 0-19 are diagnosed with cancer worldwide [1]. Advances in childhood cancer treatment have significantly improved survival rates, with more than 80% of patients receiving modern cancer therapy surviving at least 5 years beyond diagnosis, and many achieving full remission [2,3]. However, despite these improvements in medical care, childhood cancer survivors remain at risk of recurrence, secondary malignancies, chronic conditions, and functional impairments [4]. These late effects of cancer and its treatment contribute to early mortality among survivors, making ongoing health management a critical concern [5]. Physical activity (PA) is increasingly recognized as a key component in mitigating the adverse effects of cancer and its treatment [6]. Nevertheless, published data indicate that fewer than 50% of patients with pediatric cancer meet PA guidelines, highlighting a serious challenge in maintaining adequate activity levels [7]. The World Health Organization (WHO) defines PA as any bodily movement produced by skeletal muscles that requires energy expenditure, including activities performed during leisure time, transportation, and daily routines—not just structured exercise [1]. Cancer-related fatigue is a major barrier to PA, contributing to sedentary behavior and reduced mobility [8]. In addition, some patients with pediatric cancer experience physical limitations that affect their ability to perform daily activities, as observed in conditions such as clear cell sarcoma, which often affects the legs, feet, arms, hands, and torso [9]. Given these challenges, implementing interventions to counteract cancer-related physical side effects is a major priority in pediatric oncology. There is growing evidence that PA is not only safe during both the acute treatment phase and survivorship [10] but also effective in reducing cancer treatment–related side effects [11,12]. Encouraging PA in patients with pediatric cancer is therefore essential; however, existing approaches face significant barriers. Currently, hospital-based PA interventions are the most common approach, led by physiotherapists, occupational therapists, and nurses who encourage children to remain active during therapy sessions [13]. However, pediatric oncology rehabilitation services are often underutilized, with few children referred for specialized rehabilitation. Medical professionals have identified a shortage of dedicated pediatric oncology rehabilitation services, while rehabilitation specialists have highlighted the lack of appropriate environments and equipment [14]. These limitations exacerbate challenges related to organizing PA, particularly for patients who experience fatigue and mobility restrictions [7]. To address these gaps, digital technology is emerging as a valuable complement to conventional PA interventions. Technologies such as wearable sensors, augmented reality (AR), and the Internet of Things offer new approaches to engaging patients with pediatric cancer in PA and monitoring their progress. For instance, wearable devices can track step counts, gait cycles, and movement patterns in real time, providing immediate feedback on PA performance [15]. Mobile health (mHealth) apps that incorporate gamification techniques have proven effective in increasing PA by offering interactive challenges and motivational feedback, thereby helping to sustain engagement among patients with pediatric cancer [16,17]. In addition, virtual reality (VR)– and AR-based interactive digital health solutions can create immersive exercise environments, helping to overcome the lack of dedicated health care—particularly rehabilitation—spaces, while providing personalized and engaging PA experiences [18]. These technologies offer scalable, flexible, and patient-centered solutions that could enhance PA participation and adherence in pediatric oncology care [19,20]. Understanding the specific individual needs of patients with pediatric cancer is crucial for developing effective PA interventions that align with their physical capabilities and treatment constraints. This scoping review aimed to address existing research gaps by identifying conventional and digital health instruments used to monitor PA in patients with pediatric cancer during treatment. Furthermore, it examined PA monitoring methods, characterized the variables collected, and explored the potential of digital health solutions to facilitate PA engagement in patients with pediatric cancer.

The research questions guiding this review are as follows:

- What conventional and digital methods are used to monitor PA in children and adolescents with cancer?
- What variables collected by conventional and digital health instruments are used to monitor PA in children and adolescents with cancer?
- What is the applicability of different conventional and digital health instruments for monitoring PA levels in children and adolescents with cancer?
- How are digital health solutions and PA monitoring tools incorporated into interventions aimed at improving PA in children and adolescents with cancer?

In addressing these questions, the authors use the term “variables” specifically to refer to objective measurements, such as step count and gait cycles/minute, distinguishing them from subjective values obtained through self-reported questionnaires. By mapping the available PA monitoring tools and interventions, this review aims to contribute to a more comprehensive understanding of how digital solutions can support PA promotion in patients with pediatric cancer.

## Methods

### Protocol and Registration

This scoping review was conducted between April and September 2024. The review followed the Joanna Briggs Institute (JBI) methodology for scoping reviews [21,22] and was reported in accordance with PRISMA-ScR (Preferred Reporting Items for Systematic Reviews and Meta-Analyses Extension for Scoping Reviews; Multimedia Appendix 1) guidelines [23]. The 6-stage framework proposed by Arksey and O’Malley [24] was applied. The protocol was registered with the Center for Open Science [25], and the review was conducted without deviations from the registered protocol.

### Eligibility Criteria

#### Overview

Based on recommendations from the JBI [21,22] and the research questions formulated to refine the focus of the scoping review and develop an effective search strategy, we defined the eligibility criteria according to the Population, Concept, and Context framework [22].

#### Population: Children and Adolescents With Cancer

- Children and adolescents with cancer during treatment or up to 2 years after treatment. Based on existing studies, this 2-year period—including active treatment and early recovery—is used because it allows researchers to assess how PA supports the restoration of cardiorespiratory fitness and muscle strength while helping to alleviate lingering posttreatment fatigue and physical limitations [26].
- Studies including children and adolescents aged 7-19 years. This age range was selected because it corresponds to the pediatric cancer population. Younger children are generally unable to complete questionnaires or engage in PA independently, while older age groups are typically classified as adults with cancer. Children and adolescents in this age range can effectively engage in self-directed activities and structured exercise programs. Moreover, older adolescents face unique physical and psychological challenges during the transition to adulthood, making their inclusion essential for understanding the impact of PA during and after cancer treatment [6,13].

#### Concepts: Digital Health Solutions Such as Wearable Sensors, Gamification, and Mobile Apps

- Wearable sensors, AR, and gamification provide new approaches to engaging patients with pediatric cancer in PA while enabling continuous monitoring of their progress [16].
- The incorporation of AR and gamification in pediatric cancer care is justified by their demonstrated effectiveness in enhancing PA levels among children and adolescents [17,18,20].
- Digital health solutions enable objective, data-driven monitoring of PA patterns, allowing health care professionals to tailor interventions and evaluate treatment-related functional outcomes [16,20].

#### Context: Physical Activity

- PA, as defined by the WHO, encompasses bodily movement that enables individuals to perform daily activities effectively and engage in exercise [13].
- The term “physical activity” includes all forms of bodily movement performed in educational, recreational, home, or community settings, including but not limited to aerobic exercise, resistance training, flexibility, endurance activities, and stretching routines.
- Key parameters for evaluating PA include frequency, intensity, duration, type of activity, and distance covered. In pediatric cancer, assessing a child’s engagement in daily PA can provide valuable insights into recovery and health outcomes after treatment.

### Selection of Records of Evidence

Individual studies published between 2000 and March 2024 were included. The starting year of 2000 was selected to capture research conducted during the period when digital technologies began to be systematically introduced into health care and rehabilitation, marking the global transition toward digital health strategies and culminating in the WHO’s Global Strategy on Digital Health 2020-2025 [27]. The selection of records for this review was guided by predefined inclusion and exclusion criteria to ensure a comprehensive and relevant assessment of digital technologies for monitoring PA in patients with pediatric cancer. Inclusion and exclusion criteria were defined according to the Population, Concept, and Context framework (Textbox 1).

**Inclusion and exclusion criteria.**

**1. Inclusion criteria**

- Population: children and adolescents diagnosed with cancer
- Concept: digital health solutions related to physical activity (eg, augmented reality, gamification, digital tools, digital environments)
- Context: physical activity outcomes
- Setting: supervised clinical environment
- Study type: original studies with any design or data type (quantitative and qualitative)
- Publication status: published in a peer-reviewed journal
- Publication language: English
- Full-text available
- Included keywords: cancer, pediatric, oncology, exercise, monitoring, physical activity, fitness, movement, digital technologies, augmented reality, gamification, digital tools, digital environment.

**2. Exclusion criteria**

- Population: children and adolescents more than 2 years after cancer treatment
- The study does not include patients aged 7-19
- Concept: digital technologies not related to physical activity (eg, for health management)
- Context: nonphysical activity outcomes or monitoring; specific physical fitness measures
- Setting: nonclinical
- Study type: other study types (eg, protocols, narrative reviews, or systematic reviews)
- Publication status: published without peer review, dissertations, books, conference papers, letters, or editorials
- Publication language: written in a language other than English
- Full-text not available
- Excluded keywords: drug, in vitro, animal, mice, mouse, animals, bacteria, murine, rat, fish, canine, rodents, transgenic, rodent, piglets, rabbits
- Mental health outcomes are excluded unless they are mentioned in combination with a physical activity outcome.
- Duplicate data: if studies provide overlapping datasets or are part of the same project without additional insights, they should be excluded to avoid repetition.

### Information Sources and Search Strategy

A comprehensive search was conducted in ProQuest, Web of Science, EBSCO Complete, Google Scholar, ScienceDirect, Scopus, MEDLINE (PubMed), and Cochrane on April 18-19, 2024. The search strategy (Multimedia Appendix 2) combined keywords and controlled vocabulary (eg, MeSH [Medical Subject Headings] terms) related to pediatric cancer, digital technologies, PA, and outcome measurement, using Boolean operators. The final search strategy was adapted for each database. Only articles published in English were included, and gray literature was excluded. Following the database searches, all records were imported into the Rayyan AI-assisted review tool, after which the study selection process was conducted in 3 phases. First, titles and abstracts were screened, followed by full-text review to identify articles relevant to the research questions of this scoping review. In the final phase, a data extraction template was created within the Rayyan AI-assisted review tool to facilitate the systematic collection of key information from the selected articles. Given the anticipated volume of articles, all 11 coauthors participated in the selection process at each stage, with each article independently reviewed by 2 team members (AK and DB). If the Rayyan AI-assisted review tool identified a conflict regarding inclusion or exclusion, a third reviewer (GS) resolved it. Data extraction was performed independently and cross-checked to enhance accuracy. In addition, the team met regularly online to address any issues arising during the article selection phases.

### Data Charting Process

Extracted data from the reviewed articles were categorized and analyzed using a structured set of variables. Key elements of the analysis included the paper ID, first author (name and surname), journal name, and article title, providing a bibliographic foundation for the review. To contextualize the research geographically and demographically, the country of the study, study aim, and participant information (number, sex, age, diagnosis, comorbidities, treatment status, and setting) were documented. In addition, details regarding participants’ diagnosis and treatment were recorded to further clarify the clinical context. The methodology of each study was assessed, including study type (eg, cross-sectional, longitudinal), applied methods (PA monitoring instruments, questionnaires, proxy reports, digital technologies), specific tools used for monitoring, and study outcomes (objectives, data type, inclusion or empowerment, intervention effectiveness, and potential effects of monitoring methods on activity levels). In this review, the treatment period was defined as the active treatment phase, extending up to 2 years posttreatment.

### Data Synthesis and Analyses

Following the final stages of the 6-stage framework for scoping reviews proposed by Arksey and O’Malley [24], data synthesis was conducted after identifying, selecting, and charting the relevant studies. Extracted information was first organized in a structured Excel (Microsoft Corporation) matrix, enabling systematic comparison across studies. The synthesis process involved 2 main phases:

- Descriptive numerical summary, where studies were grouped by publication year, country, cancer type, participant age, treatment phase, study design, and type of PA intervention or monitoring technology. Frequency counts and distributions were used to illustrate research trends and methodological characteristics.
- Qualitative thematic synthesis, where data related to intervention characteristics, PA measurement methods, feasibility, and outcomes were coded inductively by 2 reviewers (AK and DB). Codes were iteratively compared and refined through discussion among all coauthors until consensus was reached. Emerging categories were then grouped into overarching themes, including (1) feasibility and adherence of PA monitoring, (2) barriers and facilitators to engagement, and (3) identification of digital and exercise-based interventions.

This structure enabled the systematic identification of recurring patterns, differences, and knowledge gaps across studies. Results were synthesized narratively and organized in tables to highlight key themes, relationships, and interconnections among the examined variables.

## Results

### Selection of Records of Evidence

**Figure 1:**
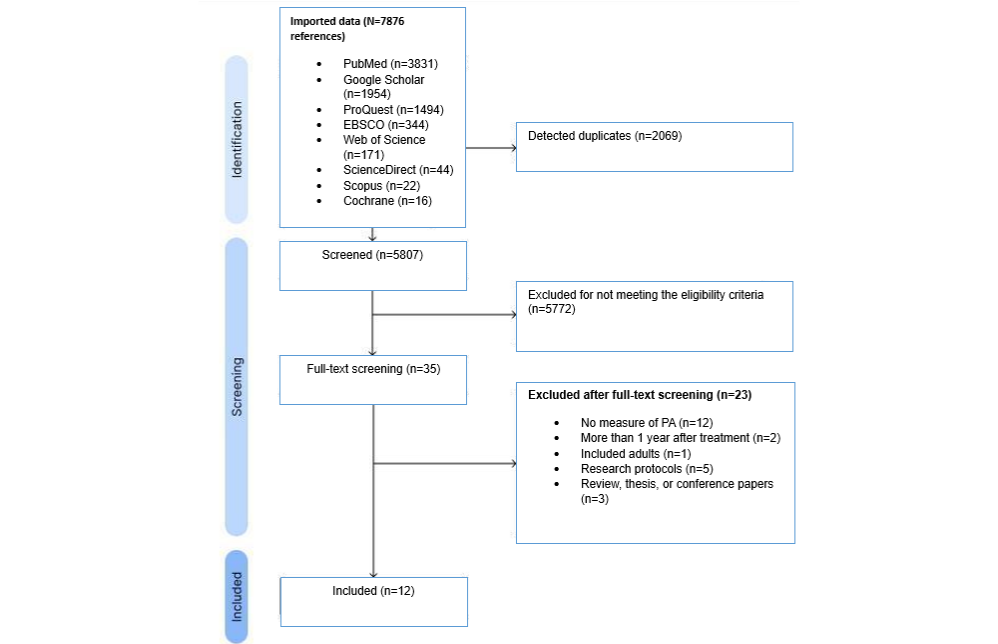
PRISMA (Preferred Reporting Items for Systematic Reviews and Meta-Analyses) flowchart of the literature search and study selection process.

Our search identified a total of 7876 records across 8 electronic databases. Of these, 2069 duplicates were removed, leaving 5807 records eligible for screening, which indicates the feasibility of our search strategy. Following the initial screening based on eligibility criteria, 5772 records were excluded, and 35 were sought for retrieval. Of these 35 records, 23 were excluded because they did not include a measure of PA, the research was conducted more than 2 years after treatment, the sample included adults, or the records were research protocols, reviews, theses, or conference papers. A total of 12 publications met all eligibility criteria and were included in this scoping review. The source and selection of evidence are presented in the PRISMA (Preferred Reporting Items for Systematic Reviews and Meta-Analyses) flow diagram (Figure 1).

### Characteristics of Included Studies

The characteristics of the 12 selected studies included in this review are summarized in Table 1. The studies encompassed various research designs, including 4 (33%) randomized controlled trials [28-31], 1 (8%) quasi-randomized trial [32], 4 (33%) cross-sectional studies [33-36], 1 (8%) multicenter cohort study [37], 1 (8%) pilot study [15], and 1 (8%) prospective observational study [38]. The included studies investigated PA levels, motor performance, and quality of life among children and adolescents diagnosed with cancer, both during and after treatment. Studies were conducted in Germany [16,27,33,37], the Netherlands [15,29,30,33], the United States [37,38], Hong Kong [36], and South Korea [32]. The interventions employed objective PA monitoring tools, such as accelerometers (Actical, Move 3, Step Watch 3, Garmin vívofit 3) and subjective self-report measures, including the Activities Scale for Kids (ASK), Pediatric Quality of Life Inventory (PedsQL)-Multidimensional Fatigue Scale, and other health-related quality-of-life instruments. Across the 12 studies, a total of 1083 participants (ages 3-20 years) were assessed, with sample sizes ranging from 25 to 482. Most participants had leukemia (acute lymphoblastic leukemia or acute myeloid leukemia) or lymphoma (non-Hodgkin lymphoma or Hodgkin), while others had solid tumors (eg, bone tumors, brain tumors, neuro-oncology cases). Some studies included mixed cancer diagnoses without specific classification. Two studies examined participants posttreatment [32,35], whereas the remaining 10 focused on patients undergoing active treatment or within the first year after completion. For example, Kang et al [32] investigated a mobile game–based healthy lifestyle program for childhood cancer survivors in South Korea, while Stössel et al [35] compared PA behaviors before, during, and after cancer treatment in Germany.

**Table 1:** Characteristics of included studies for analysis.

Author	Country	Participants’ age and setting	Disease	Research design	Objective instruments used to assess and monitor physical activity	Self-reported instruments used to assess and monitor physical activity
Gaser et al [28]	Germany	41 participants aged 4-18 years During the treatment	Acute lymphoblastic leukemia (n=25, 61%) Non-Hodgkin lymphoma (n=12, 29%) Acute myeloid leukemia (n=4, 10%)	Randomized controlled trial	The accelerometer Move 3 The Functional Activity of Daily Living Screen with everyday tasks The Motor Performance in Paediatric Oncology test	The Self-Reported Activities Scale for Kids
Gaser et al [31]	Germany	41 participants aged 4-18 years During the treatment	Acute lymphoblastic leukemia (n=25) Acute myeloid leukemia (n=4) Non-Hodgkin lymphoma (n=12) Second primary cancer (n=2)	Randomized controlled trial	The accelerometer Move 3 The Functional Activity of Daily Living Screen with everyday tasks The Motor Performance in Paediatric Oncology test	The Self-Reported Activities Scale for Kids
Braam et al [33]	The Netherlands	60 participants aged 8-18 years Treated with chemotherapy, radiotherapy, or both during or within the first year after cancer treatment	Acute lymphoblastic leukemia (n=17) Acute myeloid leukemia (n=8) Brain tumor (n=8) Hodgkin lymphoma (n=7) Bone tumor (n=7) Non-Hodgkin lymphoma (n=5) Rhabdomyosarcoma (n=3) Chronic myeloid leukemia (n=2) Others (n=3)	Cross-sectional study	Actical activity monitor	PedsQL-MFS^a^ The Participation in Sports Before the Cancer Diagnosis Questionnaire; a subscale of the Self Perception Profile Questionnaire for children aged 8-11 years and for adolescents aged 12-18 years Children’s Depression Inventory
Götte et al [34]	Germany	28 participants with a mean age of 13.8 (SD 2.8) years During cancer treatment	Leukemia (n=13) Acute lymphoblastic leukemia (n=9) Acute myeloid leukemia (n=4) Bone tumor (n=9) Ewing sarcoma (n=3) Osteosarcoma (n=6) Localized at the lower limb (n=3) Localized at trunk/upper limb (n=6) Lymphoma (n=2) Other solid tumor (n=4)	Cross-sectional study design	Step Watch 3	Physical activity questionnaire from the German Health Interview and Examination Survey for Children and Adolescents of the Robert Koch Institute
Withycombe et al [38]	The United States	65 participants aged 8-17 years Enrollment generally occurred during the first 6 months of cancer therapy, but at least 4 weeks after diagnosis and at least 3+ weeks after cancer definitive surgery (if applicable)	Leukemia/lymphoma (n=38) Solid tumor (n=16); Neuro-oncology (n=11)	Cross-sectional study design	The Garmin vívofit 3	A 9-question ecological survey PROMIS^b^
Rehorst-Kleinlugtenbelt et al [15]	The Netherlands	25 participants aged 3.1-17 years Undergoing active cancer treatment	Hematological malignancy (n=17) Solid tumors (n=8)	Cross-sectional study design	Accelerometry	N/A^c^
Mack et al [37]	The United States	482 caregivers Patients were aged 7-18 years. Participants received upfront cancer treatment, including chemotherapy and radiotherapy.	First diagnosis of cancer of any type	Multicenter cohort study	N/A	Child self-reports and caregiver proxy reports were collected for PROMIS pediatric domains, including mobility (physical functioning), pain interference, fatigue, depressive symptoms, anxiety, and psychological stress.
van Dijk-Lokkart et al [30]	The Netherlands	68 participants aged 7-18 years Patients were still receiving treatment or were within the first year after cessation of treatment	Diagnosed with any type of childhood cancer	Randomized controlled trial	Actical activity monitor	Cancer-related fatigue was assessed using both child self-report and parent proxy report versions of the PedsQL-MFS
Lam et al [36]	Hong Kong	76 participants aged 9-18 years After treatment	Leukemia (n=32, 42.1%), lymphoma (n=7, 9.2%), brain and spinal tumor (n=16, 21.1%), bone tumor (n=10, 13.2%), and others (n=11, 14.5%)	A cross-sectional study	N/A	The Chinese University of Hong Kong: Physical Activity Rating for Children and Youth Physical Activity Self-Efficacy Questionnaire The PedsQL cancer module version 3.0 PedsQL
Kang et al [32]	South Korea	51 participants aged 6-13 years All participants were childhood cancer survivors whose treatment was terminated at least 12 months prior	Childhood cancer survivors	Quasi-randomized trial	N/A	The Child Healthy Lifestyle Profile adapted for children, parents, or guardians filling out the profile
Stössel et al [35]	Germany	114 patients with cancer and 37 healthy controls between 4 and 20 years of age Completed intensive cancer treatment	Diagnosed with any type of cancer	Cross-sectional, multicenter study	N/A	Physical activity questionnaire, which is in parts based on the German Health Interview and Examination Survey for Children and Adolescents (KiGGS)
Braam et al [29]	Germany	66 participants aged 8-18 During treatment or within 12 months after treatment	N/A	Randomized controlled trial	Cardiorespiratory fitness assessed by VO_2peak_^d^ (ml kg–1 min–1) using indirect calorimetry Muscular strength was assessed using a hand-held dynamometer	Overall fatigue was assessed using the child self-report version of the PedsQL-MFS (acute version) General health-related quality of life was assessed using the Dutch self-report version of the PedsQL Behavioral problems were assessed using the Youth Self-Report Athletic competence and global self-worth were assessed using the corresponding subscales of the Self-Perception Profile Depressive symptoms were assessed using the Children’s Depression Inventory

^a^PedsQL-MFS: Pediatric Quality of Life Inventory-Multidimensional Fatigue Scale.

^b^PROMIS: Patient-Reported Outcome Measurement Information System.

^c^N/A: not applicable.

^d^VO_2peak_: peak oxygen uptake.

### PA Monitoring Methods and Obtained Variables in Patients With Pediatric Cancer

Both objective and self-reported methods were used to assess PA levels, motor performance, and fatigue in children and adolescents with cancer. Across the included studies, these 2 approaches complemented each other, providing quantitative and subjective perspectives on PA behavior and its relationship to treatment-related fatigue and recovery. Objective PA monitoring was employed in 5 studies [28,30,33,34,38], using accelerometers such as Actical, Move 3, Step Watch 3, and Garmin vívofit 3. van Dijk-Lokkart et al [30] used Actical accelerometers with a required wear time of at least 500 minutes/day to measure PA levels in children undergoing or recently completing cancer treatment. Similarly, Gaser et al [28] utilized the Move 3 accelerometer alongside motor performance tests to examine PA in patients with leukemia and lymphoma. Withycombe et al [38] used the Garmin vívofit 3 over a 4-day period to track step counts and correlate them with symptom reports. This illustrates that objective PA monitoring focused primarily on step counts and time spent in different intensity zones (light, moderate, and vigorous), reflecting a general shift toward using wearable accelerometers as a feasible and noninvasive method for tracking daily movement patterns during and after cancer treatment. In addition, subjective self-reported PA assessment methods were applied in 7 studies [28,30,33-37]. For example, the ASK, which includes 30 items across 7 subdomains (personal care, dressing, other skills, locomotion, play, standing up, and movement), and the PedsQL-Multidimensional Fatigue Scale were used. Gaser et al [28] combined ASK with motor performance testing to evaluate functional abilities such as mobility and locomotion, balance and coordination, strength and endurance, as well as fatigue and energy levels. Mack et al [37] employed caregiver-reported measures to assess mobility, pain interference, and fatigue, incorporating surveys that evaluated children’s ability to perform daily tasks, their experience of pain, and levels of physical exhaustion. Götte et al [34] assessed fatigue using the PedsQL-Multidimensional Fatigue Scale, which captures general, sleep-related, and cognitive fatigue. Additional self-report instruments, such as the Child Health Utility 9D used by Stössel et al [35], were applied to assess health-related quality of life, while structured questionnaires like the Pediatric Outcomes Data Collection Instrument, used by Braam et al [33], provided insights into mobility and participation in PAs. These self-reported instruments captured complementary domains—mobility, participation, fatigue, and quality of life—allowing researchers to contextualize accelerometer-derived data with patient-perceived outcomes and daily functional capacity. The integration of objective and self-reported measures provided a comprehensive understanding of patients’ functional status across different stages of cancer care. For comparison, while some studies focused on the active treatment phase, others, such as Kang et al [32] and Stössel et al [35], specifically examined PA behaviors in posttreatment survivors. Kang et al [32] investigated the effects of a mobile game–based healthy lifestyle program on PA levels, sedentary behavior, and overall quality of life in childhood cancer survivors in South Korea. Stössel et al [35] analyzed PA behaviors before, during, and after cancer treatment, focusing on changes in daily activity levels, mobility patterns, and engagement in sports or recreational activities. Both studies provided valuable insights into long-term functional outcomes, highlighting the impact of cancer treatment on sustained PA and overall well-being in survivors. This comparison between active treatment and posttreatment groups revealed common patterns: patients typically exhibited reduced PA levels during therapy, with gradual improvement during recovery, although many survivors continued to experience limitations in endurance and persistent fatigue. In the included studies, PA monitoring primarily focused on assessing overall activity levels using accelerometers to calculate step counts and estimate time spent in light, moderate, and vigorous activity. None of the studies conducted detailed activity recognition beyond basic actions such as walking, running, or sitting, nor did they employ multisensor systems for body motion analysis or fatigue detection. More sophisticated approaches—such as recognizing complex daily activities, detecting nuanced movement patterns, or assessing early signs of fatigue—were absent, highlighting an important gap for future research.

### Applicability of PA Monitoring Instruments in Pediatric Oncology

Overall, the studies included in this review demonstrated that PA monitoring instruments were feasible for use with patients with pediatric cancer. Feasibility was largely determined by patient adherence and device usability, both of which were reported as satisfactory in most studies. Withycombe et al [38] utilized the Garmin vívofit 3, worn on the wrist for 4 consecutive days, to measure step counts and activity patterns, observing that wearable technology effectively tracked PA, although results varied according to treatment stage and location (eg, fewer steps during hospitalization). van Dijk-Lokkart et al [30] reported good compliance with accelerometer-based PA monitoring using Actical accelerometers worn on the hip for at least 500 minutes/day over a 1-week period. Gaser et al [28] employed the Move 3 accelerometer, also positioned on the hip, with a minimum required wear time of 5 days. Some studies imposed restrictions on PA measurement, such as excluding nonambulatory patients or requiring a minimum number of valid wear days for data inclusion [34,37]. While feasibility and compliance rates were generally acceptable, the studies also highlighted important contextual and individual barriers affecting the accuracy and consistency of PA monitoring in this population. Mack et al [37] found that fatigue, treatment side effects, and hospitalization influenced step counts and overall compliance. Braam et al [29] identified psychological factors, including lack of motivation and fear of overexertion, as key barriers to PA engagement. Lam et al [36] cited time constraints and limited access to PA resources as additional challenges for participants. Despite these barriers, authors suggested that self-monitoring through wearable devices served as a motivational tool, encouraging participants to maintain PA levels. These findings suggest that while device-based PA monitoring is technically and behaviorally feasible, its effectiveness depends on addressing both physical and psychosocial barriers that influence patient participation during cancer treatment and recovery.

### Interventions for Improving PA in Pediatric Oncology

PA interventions varied across the 12 selected studies, incorporating individualized exercise programs, supervised training, and digital health tools to enhance and monitor PA levels. Intervention durations ranged from 4 weeks to 12 months, depending on study design and patient condition. Most studies reported retention rates above 80% and adherence rates ranging from 70% to 95%, indicating strong engagement with the interventions [28-30,32]. For example, Braam et al [29] reported a 92% adherence rate to supervised PA sessions, which included aerobic training, resistance exercises, and motor performance activities 2-3 times/week at moderate-to-high intensity. Similarly, Kang et al [32] reported a 90% program completion rate and high user engagement metrics (eg, daily log-ins and activity tracking) with a mobile game–based lifestyle program for childhood cancer survivors, encouraging daily movement. van Dijk-Lokkart et al [30] implemented individually tailored programs with varying intensity and duration based on the child’s physical condition, reporting over 85% adherence in most participants. Gaser et al [28] also employed individualized exercise protocols for patients with leukemia and lymphoma, focusing on moderate-intensity exercises, and reported a 100% retention rate and 88% session completion. Across these interventions, personalization of exercise type, frequency, and duration emerged as a key determinant of both adherence and safety, reinforcing the importance of individualized approaches in pediatric oncology rehabilitation.

Table 2 (also see Multimedia Appendix 3) summarizes the PA monitoring methods, variables, applicability, and interventions across the studies in pediatric oncology, providing detailed information on the tools and strategies used to assess and promote PA in this population. A summary addressing each research question, accompanied by easily interpretable tables, is presented in Multimedia Appendix 4, offering an overview of how the selected studies address the research questions.

**Table 2:** Summary of PA^a^ monitoring methods, variables, applicability, and interventions in pediatric oncology.

Reference	1. What methods are used for PA monitoring in patients with pediatric cancer?	2. What variables are collected to monitor PA in patients with pediatric cancer?	3. What is the applicability of different instruments to facilitate the PA level (monitoring) in patients with pediatric cancer?	4. What interventions are used to improve PA in patients with pediatric cancer research?
Gaser et al [28]	The Move 3 accelerometer (movisens GmbH) Self-reported questionnaire	Step count, amplitude of moderate- to-vigorous PA, body position, and wear time	Participants wore the device on the right hip during the daytime and removed the device during nighttime sleep. Records of ≥4 days of ≥8 hours/day of wear time were included. The younger participants (n=5, ages 4-7 years) felt more disturbed by the sensor on the hip. As a result, all of them refused the measurement. Because of the accelerometer’s algorithm, the PA could be calculated only for those participants aged over 7 years.	N/A^b^
Gaser et al [31]	The Move 3 accelerometer (movisens GmbH) Self-reported questionnaire	Step count	Participants wore the device on the right hip during the daytime and removed the device during nighttime sleep. Records of ≥4 days of ≥8 hours/day of wear time were included. Reasons for invalid measurements were the lack of compliance and unscheduled inpatient hospitalizations.	Exercise program–specific strength training combined with a standard care exercise program (2-3 exercise sessions/week).
Braam et al [33]	Actical accelerometer (B series; Philips Respironics Actical MiniMitter) Self-reported questionnaires	Counts/minute; the acceleration signal is summed over a specific time interval (epoch). A 15-second epoch was used in the study.	The activity monitor was attached to an elastic waist belt and worn on the left hip during daytime at waking hours (between 6:00 AM and 11:59 PM) for 4 consecutive days (Wednesday-Saturday). The device was removed while bathing. When the device was worn for less than 500 minutes/day, the measurement was considered invalid. The memory capacity of the accelerometer did not allow assessment of PA by 15-second epoch for 7 days; therefore, 4 days were used. Missing data on 3 days within the measurement week remain a limitation.	N/A
Götte et al [34]	Step Watch 3 sealed uniaxial Activity Monitor (Orthocare Innovations) Self-reported questionnaire	The volume of activity/day (gross counts/day) and intensity of activity (gross counts/minute)	The device was attached to the ankle with an elastic strap. Participants wore the activity monitor for 7 consecutive days from the morning after waking up to bedtime. Days with <8 hours of wear time were excluded. The study concluded that objective measures should be preferentially used for the assessment of PA in children and adolescents with cancer to ensure accurate and reliable data. Self-reports can complement objective measures by capturing activities or sports that are not reflected in step counts, as well as individuals’ expectations and attitudes toward exercise.	N/A
Withycombe et al [38]	The Garmin vívofit 3 accelerometer Self-reported questionnaire	Step count	Participants wore an accelerometer for 7 days. Data were included if available for at least 4 days during a defined 7-day period. Eligible days included a minimum of 10 hours of wear time between 6 AM and 10 PM. Step monitoring may serve as an objective indicator for overall symptom count, fatigue, PA, and physical function.	N/A
Rehorst-Kleinlugtenbelt et al [15]	The Actical (Philips Respironics, Mini Mitter Co, Inc)	Step count Counts/minute 15-second epoch	The device was fastened to an elastic waist belt strap and worn on the right hip. A minimum wearing time of 8 hours/day was required, with a minimum of 4 valid days a week. Parents or participants maintained a “wearing time” activity diary. The study found that accelerometry is suitable for the objective assessment of PA in children with childhood cancer during their treatment. The data gave a presentation of their PA behavior during the day. Accelerometers provide an objective assessment of PA and can be used in different kinds of patients.	N/A
Mack et al [37]	Self-reported questionnaire Caregiver proxy report	PROMIS^c^ assessments of the child’s physical function (mobility) and symptoms, including pain interference, fatigue, depressive symptoms, anxiety, and psychological stress; 5 response categories. Each question’s recall period is the past 7 days.	Our findings suggest that proxy reporting is influenced by the proxy’s personal experience of symptoms and function as well as the child’s experience. Caregivers tended to overestimate symptoms and underestimate function relative to children themselves.	N/A
van Dijk-Lokkart et al [30]	Actical accelerometer (B series; Philips Respironics Actical MiniMitter) Self-reported questionnaire Caregiver proxy report	Counts/minute, 15-second epoch	The accelerometer was worn on the hip during daytime at waking hours (between 6:00 AM and 11:59 PM) on 4 consecutive days (Wednesday-Saturday), at least 500 minutes/day over a 1-week period. Although the PedsQL-MFS^d^ has acceptable psychometric properties, including content validity, internal consistency, and responsiveness, there are inconsistent reports regarding known group validity in pediatric cancer.	Cardiorespiratory and muscle strength training twice a week for 12 weeks at a physical therapy sports center near the child’s home.
Lam et al [36]	Self-reported questionnaires	The Chinese University of Hong Kong: Physical Activity Rating for Children and Youth—score ranges from no exercise at all (0) to vigorous exercise on most days (10). PA self-efficacy score: self-confidence in PA participation, from “not sure,” “a little sure,” to “very sure.” The PedsQL cancer module version 3.0: How much of a problem was a task over the last month, from 0 to 4 (0=never, 1=almost never, 2=sometimes, 3=often, 4=almost always)?	N/A	N/A
Kang et al [32]	Self-reported questionnaire Caregiver proxy report	N/A	The healthy lifestyle program based on a mobile serious game assessed the following subdimensions: health responsibility, PA, nutrition, positive life perspective, interpersonal relations, stress management, and spiritual health. No significant effects were observed for any subdimension except PA.	A healthy lifestyle program based on a mobile serious game that promotes healthy behaviors through the completion of 26 quests, encompassing 7 subcomponents: nutrition, exercise, hygiene, interpersonal relationships, stress management, meaning of life, and health responsibility.
Stössel et al [35]	Self-reported questionnaire	Participants rated their overall level of PA on a visual analog scale, ranging from “not at all physically active” to “very physically active.” They also reported their PA across different domains, including type of activity, minutes/day, and intensity level. For analysis, total PA was calculated in minutes/week and categorized by intensity as light, moderate, or vigorous.	N/A	N/A
Braam et al [29]	Actical accelerometer (B series; Philips Respironics Actical MiniMitter) Self-reported questionnaires Caregiver proxy report	Mean counts/minute (15-second time interval)	The accelerometer was attached to an elastic waist belt and worn on the left hip during waking hours (6:00 AM to 11:59 PM). Participants wore the device for 4 consecutive days (Wednesday-Saturday). After the monitoring period, the accelerometers were returned to the research team by postal mail. Compliance with accelerometer use was low during the final study measurement week. Nonuse was primarily attributed to discomfort associated with wearing the device on a hip-mounted belt, with complaints reported particularly by girls and children who were overweight.	The 12-week intervention comprised 24 individual physical exercise sessions, consisting of two 45-minute exercise sessions/week conducted at a local physical therapy practice, and one 60-minute psychosocial training session every 2 weeks delivered to the child at the treating pediatric oncology hospital.

^a^PA: physical activity.

^b^N/A: not applicable.

^c^PROMIS: Patient-Reported Outcome Measurement Information System.

^d^PedsQL-MFS: Pediatric Quality of Life Inventory-Multidimensional Fatigue Scale.

In addition to traditional exercise and supervised rehabilitation programs, several studies integrated digital components to enhance monitoring capabilities and sustain motivation beyond clinical settings. Digital health interventions, such as Kang et al’s [32] mobile game–based program, wearable step-count monitoring [15,38], and other digital tracking tools, were used across multiple studies to assess PA levels and support motivation in patients with pediatric cancer. These programs were implemented in diverse settings, including hospitals [29,38], physical therapy centers [29,30], and home-based contexts [15,32], with supervision provided by physiotherapists, pediatric oncology specialists, or through digital applications. The inclusion of gamified and interactive elements was particularly effective in maintaining engagement among children and adolescents, consistent with evidence that motivation and enjoyment play critical roles in sustaining PA behavior during and after cancer treatment. Despite challenges such as fatigue and other treatment-related side effects, studies consistently reported that structured PA programs—including those incorporating digital elements—were associated with improved adherence and participation [30,32,38]. These digital and traditional interventions targeted a range of physical and psychosocial outcomes, with variations in duration and intensity influencing their overall effectiveness. PA intervention durations varied across studies examining effects on physical functioning [29,30,38], fitness [29,31], fatigue [30,37], quality of life [32,36], and psychological well-being [29,37]. For example, Kang et al [32] evaluated a mobile game–based intervention, which increased daily activity, improved health behaviors, and enhanced self-reported well-being. van Dijk-Lokkart et al [30] implemented a structured PA program that reduced fatigue and improved functional capacity over time. Rehorst-Kleinlugtenbelt et al [15] examined a structured physical exercise intervention delivered in both hospital and home settings, which led to increased PA levels and better adherence to exercise programs over time. By contrast, Braam et al [29] examined a structured exercise intervention delivered in a hospital and at a local physical therapy practice, which showed no significant beneficial effects on physical outcomes.

## Discussion

### Principal Findings

This scoping review identified various PA assessment instruments, including digital solutions, used to monitor PA in patients with pediatric cancer. Self-reported questionnaires remain the most frequently used instruments due to their accessibility and cost-effectiveness [28-30,32-39]. While these instruments provide valuable insights from the patient’s perspective, their reliability is limited by recall bias and motivational factors, making them inherently subjective. Although widely utilized, self-reported questionnaires have been increasingly complemented by accelerometry, which provides empirical, quantifiable measurements of PA [38,39]. Self-reported surveys are often favored because they are inexpensive, easy to administer, and adaptable to different age groups, making them feasible even in larger study settings. Self-reported questionnaires also provide important patient-centered insights by capturing subjective experiences such as fatigue, pain interference, and broader quality-of-life outcomes (eg, Götte et al [34], Mack et al [37], Stössel et al [35], Braam et al [33]). However, their accuracy is limited by recall bias, motivational influences, and social desirability, which may distort reported activity levels. While self-reported surveys offer valuable perspectives on how children and adolescents perceive their PA and related symptoms, they are less reliable for accurately quantifying activity levels. This underscores the importance of complementing self-reported surveys with objective monitoring tools to achieve a more comprehensive assessment of PA in pediatric cancer. Self-reported questionnaires remain widely used due to their low cost, ease of administration, and ability to capture subjective patient experiences that objective measures may not assess. These instruments are also feasible for large-scale studies and adaptable to different age groups. However, subjective methods are prone to bias, social desirability, and motivational influences, which can affect the accuracy of data outcomes. In summary, self-reported questionnaires demonstrate lower reliability in quantifying PA levels compared with objective instruments such as accelerometers.

Accelerometer-based approaches provided objective data such as step counts, wear-time compliance, and time spent at different activity intensity levels (eg, Actical in van Dijk-Lokkart et al [30], Garmin vívofit 3 in Withycombe et al [38], Move 3 in Gaser et al [28]). In some studies, accelerometry was combined with motor performance testing to assess balance, coordination, strength, and endurance [28]. Other studies extended the focus to participation in recreational or social activities, linking activity patterns to broader dimensions of daily life [33,34]. Overall, accelerometers appear to be practical and feasible tools for objectively monitoring PA in patients with pediatric cancer. Accelerometers provide reliable, quantifiable data and are generally well-tolerated by participants; however, their usefulness is limited by the need for compliance, inability to capture complex or context-specific activities, and the lack of standardized protocols across studies. When used alongside complementary instruments, accelerometers can be a valuable component of PA monitoring in both research and clinical practice. Despite growing interest in interventions using digital tools, this review found that only 1 study employed a mobile game–based approach for PA tracking in patients with pediatric cancer [32], which also functioned as a mobile engagement tool. In this study, children participated in a serious game requiring completion of 26 quests, including 7 subelements (nutrition, exercise, hygiene, interpersonal relationships, stress management, meaning of life, and health responsibility), to promote a healthy lifestyle [32]. This review demonstrated the potential of digital solutions to integrate PA monitoring with the promotion of healthy lifestyle habits during cancer treatment in clinical settings. The selected studies also highlighted the limited research outcomes available to date. While digital tools may be feasible and applicable, their broader implementation in pediatric oncology remains largely unexplored.

The integration of PA monitoring with patient engagement strategies for behavior change, such as gamification and AR, holds promise for enhancing both data accuracy and patient motivation [18,32]. This highlights a research gap where interactive digital solutions could play a pivotal role in optimizing PA interventions for patients with pediatric cancer. While prior studies have extensively examined the effectiveness of digital technology in promoting PA [15,31,32,38], only a few have explored how PA monitoring itself can facilitate PA participation and adherence [30,32,38]. Although most studies relied on accelerometers and questionnaires, a gap remains regarding the use of AR and gamification specifically for PA monitoring in pediatric oncology. Studies employed a variety of methods to promote PA in children and adolescents with cancer, including self-reported questionnaires, wearable accelerometers, and digital game–based tools. Self-reported questionnaires, such as the ASK used by Gaser et al [28] and the PedsQL-Multidimensional Fatigue Scale applied by Götte et al [34], Mack et al [37], and others [33,36], captured subjective experiences of mobility, fatigue, pain, and quality of life, providing valuable patient-centered insights, though they are limited by recall bias and motivational factors. Wearable devices, including Actical accelerometers in van Dijk-Lokkart et al [30], Move 3 in Gaser et al [28], and Garmin vívofit 3 in Withycombe et al [38], offered objective, continuous measures of step counts, activity intensity, and motor performance, enhancing accuracy and enabling longitudinal monitoring, but they may fail to capture complex or context-specific activities and depend on participant compliance. Digital interventions, such as Kang et al’s [32] mobile game–based program, combined PA tracking with engagement and health education, supporting adherence and motivation; however, evidence remains limited, and these tools are largely unvalidated. Hybrid approaches integrating subjective and objective measures, as applied in several studies [32,38], show promise for providing a multidimensional understanding of PA and potentially improving adherence, though they require greater resources and supervision. Overall, these findings highlight the need to further explore innovative interventions that implement digital tools designed to be both engaging and user-friendly for patients with pediatric cancer. For instance, AR-based interactive games have been proposed as a promising approach, as they may enable real-time monitoring and provide personalized support for daily PA. Such technologies have the potential to offer health care professionals accurate information about a patient’s daily activity level and enhance motivation to remain active during different treatment stages. Further research is required to evaluate the impact of digital tools on physical and mental health outcomes in patients with pediatric cancer, while also addressing the limitations of current methods highlighted in this review.

### Implication for Practice

This review underscores the need for a standardized approach to PA monitoring in patients with pediatric cancer, as current practices are heterogeneous and often lack consistency. While innovative digital solutions, such as AR and gamification, may offer promising approaches to enhance motivation and adherence to PA interventions, their applicability and effectiveness in pediatric oncology remain largely unexplored and require further investigation.

Additionally, this review highlighted the need for a standardized approach to PA monitoring in patients with pediatric cancer. The diversity of accelerometers [28-34,38] and self-report questionnaires [28-38] underscores the necessity for unified guidelines to ensure consistency in data collection and interpretation. Developing standardized guidelines for PA assessment would enhance comparability across studies and support the integration of digital monitoring solutions into clinical practice.

### Limitations

This review has several limitations. It included only articles published in English, which may have resulted in the exclusion of relevant research published in other languages. Additionally, the focus on children and adolescents aged 7-19 years who were undergoing treatment or within 2 years posttreatment may have limited insights into long-term PA outcomes beyond this period. Furthermore, heterogeneity in study designs, PA measurement tools, and intervention methodologies posed challenges for direct comparisons. The aim of this review was not to evaluate the validity or reliability of the selected instruments but to map the available measurement methods and digital solutions used to monitor PA in patients with pediatric cancer. The limited adoption of interactive digital solutions highlights a critical research gap, underscoring the need for further studies to evaluate their feasibility, appropriateness, and long-term impact on PA engagement in this population.

### Future Directions

Our findings indicate that future research should focus on exploring how PA monitoring can actively facilitate PA participation in patients with pediatric cancer. While numerous studies have examined PA monitoring tools, only a few have assessed their impact on promoting PA. Further investigation is also needed into the use of AR-based interventions and gamification for PA monitoring in pediatric cancer. Among the 12 included studies, none involved an AR-based intervention, and the only digital engagement tool identified was a gamified mobile game by Kang et al [32]. AR represents an unexplored but promising area for future research in pediatric oncology rehabilitation. Interactive digital interventions have the potential to both measure and enhance PA engagement by providing real-time feedback and motivation [18]. Evaluating patient acceptance, clinical integration, and long-term adherence to such technologies will be crucial to optimizing their effectiveness. Furthermore, the absence of standardized PA monitoring protocols highlights the need for unified guidelines for digital monitoring solutions tailored to patients with pediatric cancer. Establishing consistent assessment criteria would improve comparability across studies and facilitate the integration of digital monitoring solutions into routine clinical practice.

### Conclusions

Despite the growing need to monitor and promote PA in patients with pediatric cancer, opportunities for interactive and engaging PA monitoring remain limited. The findings of this scoping review indicate an emerging body of literature on digital health technologies, including wearable sensors such as accelerometers, mHealth apps, and gamification, which are being explored for PA assessment and engagement. Future research should further investigate the purpose, scope, and integration of digital health technologies to facilitate interactive and personalized approaches for effectively monitoring and enhancing PA in both clinical and home-based settings for patients with pediatric cancer.

## Appendix

Multimedia Appendix 1 PRISMA-ScR checklist.

Multimedia Appendix 2 Search strategy.

Multimedia Appendix 3 Detailed summary of physical activity monitoring methods, variables, applicability, and interventions in pediatric oncology.

Multimedia Appendix 4 Research questions.

## Abbreviations

AR: augmented reality
ASK: Activities Scale for Kids
JBI: Joanna Briggs Institute
MeSH: Medical Subject Headings
mHealth: mobile health
PA: physical activity
PedsQL: Pediatric Quality of Life Inventory
PRISMA: Preferred Reporting Items for Systematic Reviews and Meta-Analyses
PRISMA-ScR: Preferred Reporting Items for Systematic Reviews and Meta-Analyses Extension for Scoping Reviews
VR: virtual reality
WHO: World Health Organization
