# Supplementary material for: Measurements and Digital Technology Solutions to Monitor Physical Activity in Patients With Pediatric Cancer: Scoping Review
Source: JMIR Cancer. 2026 Jan 29;12:e73889. doi: 10.2196/73889 (PMC12902754; doi:10.2196/73889)
Supplement: Multimedia Appendix 2 [file cancer_v12i1e73889_app2.docx]

**Appendix 1**: Database search details.

Database limit: 2000 to March 2024, English.

**Exclusion criteria:** Population: children and adolescents more than 2 years after cancer treatment; The study does not include patients aged 7-19;

Concept: digital technologies not related to PA (e.g. for health management); Context: non-physical activity outcomes or monitoring; specific physical fitness measures; Setting: Nonclinical; Study type: other study types (e.g. protocols, narrative reviews, or systematic reviews); Publication status: published without peer review, dissertations, books, conference papers, letters, or editorials; Publication language: written in a language other than English; Full-text not available; Excluded keywords: drug, in vitro, animal, mice, mouse, animals, bacteria, murine, rat, fish, canine, rodents, transgenic, rodent, piglets, rabbits; Mental health outcomes are excluded unless they are mentioned in combination with a physical activity outcome; Duplicate data: If studies provide overlapping data sets or are part of the same project without additional insights, they should be excluded to avoid repetition.

| **Database** | **Search terms** | **Number of studies** |
| --- | --- | --- |
| ProQuest | ("Pediatric oncology patients" OR "Pediatric cancer patients" OR "adolescent cancer patients") AND ("Digital environment" OR "Digital Solutions" OR "Digital Tools" OR "Augmented Reality" OR AR OR "Gamification") AND ("Physical Activity" OR PA OR Mobility OR Fitness OR Exercise OR "Sedentary behaviour" OR Sport OR Movement) AND ("Self reported" OR "Objective measurements" OR Measurement OR Assessment OR "Outcome measures" OR "Patient feedback" OR PROM OR PREM OR Monitoring OR "Patient reported outcomes" OR "Patient Reported outcome measures" OR "Patient reported experience measures") | 1494 |
| the Web of Science | ALL=(("pediatric oncology patients" OR "paediatric oncology patients" OR "pediatric cancer patients"))  AND ALL=("physical activity")  AND ALL=("digital environment" OR "digital solutions" OR "digital tools" OR "augmented reality" OR AR OR gamification)  AND ALL=("self reported" OR "objective measurements" OR measurement OR assessment OR "outcome measure" OR "patient feedback" OR PROM OR PREM OR monitoring) | 171 |
| EBSCO Complete | ("pediatric oncology patients" OR "paediatric oncology patients" OR "pediatric cancer patients" OR "paediatric cancer patients" OR "adolescent cancer patients")  AND ("physical activity")  AND ("digital environment" OR "digital solutions" OR "digital tools" OR "augmented reality" OR AR OR gamification)  AND ("self reported" OR "objective measurements" OR measurement OR assessment OR "outcome measure" OR "patient feedback" OR PROM OR PREM OR monitoring OR "patient reported outcome measures" OR "patient reported experience measures") | 344 |
| Google Scholar | 1) With digital/tech component (captures all your digital, AR, and gamification variants):  ("pediatric oncology patients" OR "paediatric oncology patients" OR "pediatric cancer patients" OR "paediatric cancer patients" OR "adolescent cancer patients")  "physical activity"  ("digital environment" OR "digital solutions" OR "digital tools" OR "augmented reality" OR AR OR gamification)  ("self reported" OR "self-reported" OR "objective measurements" OR "objective measurement" OR measurement OR assessment OR "outcome measure" OR "outcome measures" OR "patient feedback" OR PROM OR PREM OR monitoring OR "patient reported outcome measures" OR "patient reported experience measures")  2) Measures-focused (no digital terms; covers your “Patient + Physical + Measures” set):  ("pediatric oncology patients" OR "paediatric oncology patients" OR "pediatric cancer patients" OR "paediatric cancer patients" OR "adolescent cancer patients")  "physical activity"  ("self reported" OR "self-reported" OR "objective measurements" OR "objective measurement" OR measurement OR assessment OR "outcome measure" OR "outcome measures" OR "patient feedback" OR PROM OR PREM OR monitoring OR "patient reported outcome measures" OR "patient reported experience measures") | 1954 |
| Science Direct | ("pediatric oncology patients" OR "paediatric oncology patients" OR  "pediatric cancer patients" OR "paediatric cancer patients" OR  "adolescent cancer patients")  AND  ("physical activity" OR PA OR mobility OR fitness OR exercise OR sport OR movement OR "sedentary behavior")  AND  ("digital environment" OR "digital solution" OR "digital tool" OR "digital technolog" OR  "augmented reality" OR AR OR gamification OR "game element")  AND  ("self report" OR "objective measur" OR measur OR assess OR "outcome measur" OR  "patient feedback" OR PROM OR PREM OR monitor OR  "patient reported outcome" OR "patient reported experience") | 44 |
| Scopus | ALL ( ( "Pediatric oncology" ) OR ( "Pediatric cancer" ) OR ( "Adolescent cancer" ) AND ( "Digital environment" ) OR ( "Digital solutions" ) OR ( "Digital tools" ) OR ( "Augmented reality" ) OR ( "AR" ) OR ( "Gamification" ) OR ( "Game elements" ) ) AND ( ( "Physical Activity" ) OR ( "PA" ) OR ( "Mobility" ) OR ( "Sedentary behavior" ) OR ( "Fitness" ) OR ( "Exercise" ) OR ( "Sport" ) OR ( "Movement" ) ) AND ( ( "Self reported" ) OR ( "Objective measurements" ) OR ( "Measurement" ) OR ( "Assessment" ) OR ( "Outcome measure" ) OR ( "Patient feedback" ) OR ( "PROM" ) OR ( "PREM" ) OR ( "Monitoring" ) OR ( "Patient reported outcomes" ) OR ( "Patient Reported Outcome Measures" ) OR ( "Patients Reported Experience Measures" ) ) AND ( LIMIT-TO ( DOCTYPE , "ar" ) OR LIMIT-TO ( DOCTYPE , "re" ) ) AND ( LIMIT-TO ( LANGUAGE , "English" ) ) | 22 |
| MEDLINE (PubMed) | (  "pediatric oncology patients"[tiab] OR "paediatric oncology patients"[tiab] OR  "pediatric cancer patients"[tiab] OR "paediatric cancer patients"[tiab] OR  "adolescent cancer patients"[tiab]  )  AND  (  "physical activity"[tiab] OR physical activ[tiab]  )  AND  (  "digital environment"[tiab] OR digital solution[tiab] OR digital tool[tiab] OR  digital technolog[tiab] OR "augmented reality"[tiab] OR "Augmented Reality"[Mesh] OR  AR[tiab] OR gamification[tiab] OR "virtual reality"[tiab] OR "Virtual Reality"[Mesh]  )  AND  (  "self reported"[tiab] OR "self-reported"[tiab] OR  "objective measurement"[tiab] OR "objective measurements"[tiab] OR  measur[tiab] OR assess[tiab] OR  "outcome measure"[tiab] OR "outcome measures"[tiab] OR  "patient feedback"[tiab] OR PROM[tiab] OR PREM[tiab] OR monitor[tiab] OR  "patient-reported outcome"[tiab] OR "patient-reported outcomes"[tiab] OR  "patient reported outcome"[tiab] OR "patient reported outcomes"[tiab] OR  "patient-reported experience"[tiab] OR "patient-reported experiences"[tiab] OR  "patient reported experience"[tiab] OR "patient reported experiences"[tiab]  ) | 3831 |
| Cochrane | ("pediatric oncology patients" OR "paediatric oncology patients" OR  "pediatric cancer patients" OR "paediatric cancer patients" OR  "adolescent cancer patients")  AND  ("physical activity" OR "PA" OR "mobility" OR "fitness" OR "exercise" OR "sport" OR "movement" OR "sedentary behaviour" OR "sedentary behavior")  AND  ("digital environment" OR "digital solution" OR "digital tool" OR "digital technolog" OR  "augmented reality" OR AR OR gamification OR "game element")  AND  ("self reported" OR "self-reported" OR "objective measurement" OR measur OR assess OR  "outcome measure" OR "patient feedback" OR PROM OR PREM OR monitor OR  "patient reported outcome" OR "patient reported experience") | 16 |
