# Supplementary material for: Measurements and Digital Technology Solutions to Monitor Physical Activity in Patients With Pediatric Cancer: Scoping Review
Source: JMIR Cancer. 2026 Jan 29;12:e73889. doi: 10.2196/73889 (PMC12902754; doi:10.2196/73889)
Supplement: Multimedia Appendix 3 [file cancer_v12i1e73889_app3.docx]

*Table 2. Summary of Physical Activity Monitoring Methods, Variables, Applicability, and Interventions in Pediatric Oncology (Multimedia Appendix 4).*

| **Paper ID, author, year** | **1. What methods are used for physical activity monitoring in pediatric cancer patients?** | **2. What variables are collected to monitor PA in pediatric cancer patients?** | **3. What is the applicability of different instruments to facilitate PA level (monitoring) in pediatric cancer patients?** | **4. What interventions are used to improve PA in pediatric cancer patients?research?** |
| --- | --- | --- | --- | --- |
| 1.  [27] Dominik Gaser et al 2022 | The accelerometer, self-reported questionnaire.  Move 3 accelerometer (movisens GmbH, Karlsruhe, Germany).  It assesses triaxial movement accelerations with a frequency of 64 Hz and a measurement range of ±16 g. The raw sensor data are based on 3D acceleration, temperature and atmospheric air pressure. The inclination obtained from the acceleration was used to classify body position. | Step count, amplitude of moderate- to-vigorous PA (MVPA), body position and wear time.  Wear time was calculated in 30 s intervals. | The participants wore the device on the right-hip side during daytime and removed the device for night-time sleep. Records of ≥4 days of ≥8 h/day of wear time were included.  The younger participants (n = 5, ages 4–7 years) felt more disturbed by the sensor on the hip. As a result, all of them refused the measurement. Because of the accelerometer's given algorithm, the PA could be calculated only for those participants >7 years. |  |
| 2.  [39]  Dominik Gaser et al 2022 | The accelerometer, self-reported questionnaire.  Move 3 accelerometer (movisens GmbH, Karlsruhe, Germany). | Step count. | The participants wore the device on the right-hip side during daytime and removed the device for night-time sleep. Records of ≥4 days of ≥8 h/day of wear time were included.  Reasons for invalid measurements were the lack of compliance and unscheduled inpatient hospitalizations. | Exercise program- specific strength  training combined with a standard care exercise program (2–3 exercise sessions per week). |
| 3.  [32] Katja I. Braam et al 2016 | The accelerometer, self-reported questionnaires..  Actical accelerometer,  B series, Philips Respironics Actical MiniMitter, Murrysville, PA, USA.  The receiver operating characteristic curves were 0.85, 0.93, and 0.95 for a sedentary to light, light to moderate, and a moderate to vigorous activity level, respectively. | Counts per minute (cpm). The acceleration signal is summed over a specific time interval (epoch). A 15-s epoch was used in the study.  Cpm range to define the different activity intensities: sedentary status corresponds with an activity count of less than 100 cpm, light activity with 100–1599 cpm, and moderate activity with 1600–4760 cpm, and 4760 or more cpm was considered as a vigorous activity level.  SB (sedentary behavior), defined as a cpm below 100 was presented as mean minutes sedentary (out of 1080 measured min/day) and as accelerometer-based sedentary bouts. Sedentary bouts were defined as periods of at least 5, 10, 20, 30, and 60 min of SB. | The activity monitor was attached to an elastic waist belt and worn on the left hip during daytime at waking hours (between 6:00 a.m. and 11:59 p.m.) for four consecutive days (Wednesday–Saturday).The device was removed while bathing.   When the device was worn <500 min/day, the measurement was considered invalid.  The memory capacity of the accelerometer did not allow assessment of PA by 15-s epoch for a length of 7 days, therefore 4 days were used. Missing data of 3 days within the measurement week is a limitation. |  |
| 4.  [33] Miriam Gotte et al 2017 | The accelerometer, self-reported questionnaire.  Step Watch 3™ sealed uniaxial Activity Monitor, SAM, Orthocare Innovations, Mountlake Terrace, WA 98043, USA. | The volume of activity per day (gcs per day) and intensity of activity  (gcs per minute). Gcs = gait cycles (2 steps) per time interval (1 min).  50 gcs/min (= 100 steps/min) is a threshold for moderate to vigorous activity levels and 20 gcs/min- threshold for continuous walking. | The device was attached to the ankle with an elastic strap.Participants wore the SAM for 7 consecutive days from morning after waking up until bedtime.   Days with <8h of wear time were excluded.  As a conclusion- objective measures should be preferentially used for the assessment of PA in children and adolescents with cancer to ensure accurate and reliable data. Self-reports can complement objective measures by assessing categories of activities or sports that do not involve step counts, as well as expectations and attitudes toward exercise. |  |
| 5.  [28] Janice S. Withycombe et al 2022 | The accelerometer, self-reported questionnaire.  The Garmin VivoFit® 3 accelerometer. | Step count. | Participants wear an accelerometer for 7 days. Data were included if available for at least 4 days during a defined 7-day period. Eligible days included a minimum of 10 hours of wear time between the hours of 6am and 10pm.   This monitor was selected for its ease of use, long battery life (one year), robust memory (30 days), water resistance, small size of the monitor unit, availability of both adult/adolescent and child sized wrist bands, and for its low cost (< $90 per monitor).  Missing data was higher than anticipated primarily related to non-wear time and difficulty retrieving (syncing) data from a commercial device.  Step monitoring may serve as an objective indicator for overall symptom count, fatigue, physical activity and physical function |  |
| 6.  [16] Willem Peter Bekkering et al 2019 | The accelerometer.  The Actical, Philips Respironics, Mini Mitter Co, Inc., Bend, OR. | Step count, count per minute, 15s epoch. Data in cpm were defined by averaging the four 15 s epoch counts over each minute [40] and (iv) daily number of steps.  SB was defined as < 100 cpm. | The device was fastened to an elastic waist belt strap,worn on the right hip. The minimum wearing time of 8 h per day was required, and the minimum number of 4 valid days a week.    Parents or participants kept up a ‘wearing time’ activity diary. The study found that accelerometery seems to be suitable for the objective assessment of PA in children with childhood cancer during their treatment. The data give a presentation of their PA behaviour during the day. Accelerometers provide an objective assessment of PA and can be applied with different kinds of patients.   Choosing the Actical also gave some limitations, e.g. SB could be overestimated because during daytime children did not wear the Actical when taking naps or showers. |  |
| 7.  [34] Jennifer W. Mack et al 2020 | Self-reported questionnaire, caregiver-proxy  report. | PROMIS- assessments of the child’s physical function (mobility) and symptoms, including pain interference, fatigue, depressive symptoms, anxiety, and psychological stress; 5 response categories. Each question’s recall period is the past 7 days. | Our findings suggest that proxy reporting is influenced by the proxy’s personal experience of symptoms and function as well as the child’s experience. Caregivers tended to overestimate symptoms and underestimate function relative to children themselves. |  |
| 8.  [30] Elisabeth M. Van Dijk-Lokkart et al 2019 | The accelerometer, self-reported questionnaire, caregiver-proxy  Report.  Actical activity monitor; B series, Philips Respironics Actical Mini Mitter, Co Inc., USA. | Count per minute, 15s epoch.  Physical activity of <100 CPM was considered sedentary, <900 CPM as light activity, <2200 CPM as moderate activity, and ≥2200 CPM as vigorous activity. | Accelerometer was worn on the hip during daytime at waking hours (between 6:00 am and 11:59 pm) on four consecutive days (Wednesday Saturday), at least 500 minutes per day over a one-week period.  . Although the PedsQL-MFS has acceptable psychometric properties, including content validity and internal consistency and responsiveness, there are inconsistent reports regarding known group validity in pediatric cancer. | Cardiorespiratory and muscle strength training twice a week, 12 weeks at a physical therapy sports center near the child's home. |
| 9.  [36] Katherine K.W. Lam et al  2016 | Self-reported questionnaires. | The Chinese university of Hong Kong: physical activity rating for children and youth- score ranges from no exercise at all (0) to vigorous exercise on most days (10). Physical activity self-efficacy- score self-confidence in participation in PA, from ‘not sure’, ‘a little sure’, to ‘very sure’. The pediatric quality-of-life inventoryTM (PedsQLTM) cancer module v. 3.0- how much a problem was a task over last month, from  0 to 4 (0 ¼ never, 1 ¼ almost never, 2 ¼ sometimes, 3 ¼ often, 4 ¼ almost always). |  |  |
| 10.  [31] Kyung-ah Kang et al 2024 | Self-reported questionnaire, caregiver-proxy  report. |  | A healthy lifestyle program based on a mobile serious game (HLP-MSG)-observed such sub-dimensions as health responsibility, PA, nutrition, positive life perspective, interpersonal relations, stress management, spiritual health. No significant effect was observed on any of the sub-dimensions except physical activity. | HLP-MSG: Healthy lifestyle program based on a mobile serious game that promote a healthy lifestyle by solving 26 quests, including seven sub-elements (nutrition, exercise, hygiene interpersonal relationships, stress management, meaning of life, and health responsibility). |
| 11.  [35] Sandra Stossel et al 2020 | Self-reported questionnaire. | On a visual analogue scale (VAS), participants were asked how physically active they were- from “not at all physically active” to “very physically active”. And how physically active they were in the various domains, type of activity,minutes per day and the intensity level. The amount of physical activity in minutes per week and intensity levels light (LPA), moderate (MPA) and vigorous (VPA) were used for analysis of the results. |  |  |
| 12.  [29] Katja I. Braam et al 2018 | The accelerometer,  self-reported questionnaires,  caregiver-proxy  report.  Actical accelerometer, B series, Philips Respironics Actical MiniMitter, Murrysville, PA, USA. Mean counts per minute is a physical activity score including horizontal, vertical and depth motion scores in one end score; higher scores indicate more activity. | Mean counts per minutes, 15s time-interval. | The accelerometer was attached to an elastic waist belt, and worn on the left hip during daytime at waking-hours (between 6:00 am and 11:59pm). It was worn on four consecutive days: Wednesday to Saturday. After wearing, participants sent the accelerometers back to the research team by postal mail.   The low number of patients wore the PA monitor during the final study measurement week. The reason for not wearing the accelerometer was related to the discomfort of wearing the monitor through a belt on the hip. Complaints especially came from girls and overweighed children. | The 12-week intervention consisted of 24 individual physical exercise sessions (two 45-min physical exercise sessions per week  at a local physical therapy practice and one 60-min psychosocial training session once every 2 weeks for the child in the treating pediatric oncology hospital ). |
