# Supplementary material for: Measurements and Digital Technology Solutions to Monitor Physical Activity in Patients With Pediatric Cancer: Scoping Review
Source: JMIR Cancer. 2026 Jan 29;12:e73889. doi: 10.2196/73889 (PMC12902754; doi:10.2196/73889)
Supplement: Multimedia Appendix 4 [file cancer_v12i1e73889_app4.pdf]

Q1. Methods used for PA monitoring — ranked by frequency

Rank	Tool (Device)	Frequency (studies)	Typical placement	Wear protocol (examples)
1	Actical	2	hip; right hip	≥500 min/day over one week; 15 s epoch	≥8 h/day; ≥4 valid days/week; 15 s epoch
2	Move 3 accelerometer	2	hip	7 consecutive days (outpatient)	≥5 days wear time
3	Accelerometer (unspecified)	1	-	15 s interval; mean counts/min
4	Actical activity monitor	1	left hip (waist belt)	4 consecutive days (Wed–Sat), daytime 6:00–23:59
5	Garmin VivoFit 3	1	-	7 days
6	StepWatch 3 (SAM)	1	ankle	7 consecutive days, waking to bedtime

Q2. Variables collected to monitor PA — ranked by frequency

Rank	Variable	Frequency (studies)
1	Step count	4
2	15 s epoch	2
3	Counts per minute	2
4	15 s interval	1
5	Activity intensity (gcs/min)	1
6	Body position	1
7	Counts per minute (cpm)	1
8	Gait cycles per day (gcs/day)	1
9	Mean counts per minute	1
10	Minutes/week by intensity (LPA, MPA, VPA)	1
11	MVPA	1
12	Wear time	1

Q3. Applicability of instruments (implementation details)

Study	Instrument	Placement	Wear protocol	Epoch/Interval	Notes
Gaser et al., 2022 [27]	Move 3 accelerometer	hip	≥5 days wear time	-	-
Gaser et al., 2022 [39]	Move 3 accelerometer	-	7 consecutive days (outpatient)	-	-
Braam et al., 2015 [32]	Actical activity monitor	left hip (waist belt)	4 consecutive days (Wed–Sat), daytime 6:00–23:59	-	-
Götte et al., 2017 [33]	StepWatch 3 (SAM)	ankle	7 consecutive days, waking to bedtime	-	-
Withycombe et al., 2022 [28]	Garmin VivoFit 3	-	7 days	-	-
Rehorst-Kleinlugtenbelt et al., 2019 [16]	Actical	right hip	≥8 h/day; ≥4 valid days/week; 15 s epoch	15 s	-
Van Dijk-Lokkart et al., 2019 [30]	Actical	hip	≥500 min/day over one week; 15 s epoch	15 s	Caregiver proxy used
Braam et al., 2018 [29]	Accelerometer (unspecified)	-	15 s interval; mean counts/min	15 s	Caregiver proxy used

Q4. Interventions to improve PA (4 studies)

Study	Intervention type	Frequency	Duration	Setting/Notes
Gaser et al., 2022 [39]	Specific strength training + standard care exercise	2–3 sessions/week	not stated	
Van Dijk-Lokkart et al., 2019 [30]	Cardiorespiratory + muscle strength training at PT sports center	2×/week	12 weeks	
Kang et al., 2024 [31]	Healthy Lifestyle Program based on a Mobile Serious Game (HLP-MSG)	26 quests incl. 7 sub-elements	not stated	No significant effect except PA sub-dimension
Braam et al., 2018 [29]	Combined: 24 individual exercise sessions + psychosocial training	2×45 min/week exercise; 1×60 min psychosocial every 2 weeks	12 weeks	

Coverage matrix of research questions by study

Study	Q1 (methods)	Q2 (variables)	Q3 (applicability)	Q4 (interventions)
Gaser et al., 2022 [27]	✓	✓	✓	
Gaser et al., 2022 [39]	✓	✓	✓	✓
Braam et al., 2015 [32]	✓	✓	✓	
Götte et al., 2017 [33]	✓	✓	✓	
Withycombe et al., 2022 [28]	✓	✓	✓	
Rehorst-Kleinlugtenbelt et al., 2019 [16]	✓	✓	✓	
Mack et al., 2020 [34]	✓	✓		
Van Dijk-Lokkart et al., 2019 [30]	✓	✓	✓	✓
Lam et al., 2016 [36]	✓	✓		
Kang et al., 2024 [31]	✓		✓	✓
Stössel et al., 2019 [35]	✓	✓		
Braam et al., 2018 [29]	✓	✓		✓
